# The Impact of Scaffolding on Academic Achievement in Undergraduate Emergency Language Courses

**DOI:** 10.1007/s10936-026-10254-9

**Published:** 2026-06-01

**Authors:** An Zhu, Jian-Hong Ye

**Affiliations:** 1 Higher Vocational Education Research Institute, Yunnan Vocational College of Transportation, No. 9, Ninghu West Road, Kunming, China; 2 Faculty of Education, Beijing Normal University, No. 19, Xinjiekouwai St, Haidian District, Beijing, China

**Keywords:** Constructivist learning theory, Expected value theory, Learning achievement, Perceived teacher support, Quasi-experiment, Scaffolding, Self-regulated learning

## Abstract

The COVID-19 epidemic as a global public health emergency has brought unprecedented attention to emergency languages. Emergency language proficiency is an essential component of national language proficiency, making the development of emergency language service personnel particularly important. Therefore, in this study, 225 students of teaching Chinese to speakers of other languages at a Chinese university were invited to participate in a 8-week quasi-experimental design of a control group (traditional teaching) and an experimental group (scaffolded instruction) to conduct an emergency language course. Based on the Expected Value Theory of Achievement Motivation, this study proposed eight research hypotheses and constructed a research model to understand the effects of students' perceived teacher support on self-regulated learning (SRL) and learning achievement in a scaffolding model.

## Introduction

The international attention to the adaptability to disasters around the world is increasing (Cadwell & O'Brien, 2016), language translation is gradually becoming a key element in disaster prevention and response in disaster management (O'Brien, 2019). After the outbreak of COVID-19, emergency language services as a part of language life have been incorporated into the national emergency governance mechanism (Xi & Liu, 2020), and emergency language competence also became a component of national language competence (Zeng, 2022). The Ministry of Education (2020) issued the “Three Year Action Plan of the National Emergency Language Service Team (2023–2025),” stating that carry out emergency language service education and promote the construction of emergency language service disciplines and majors. However, there is a dearth of research on emergency language services talent, revealing the lack of a system for emergency language services talent construction in China. There is an urgent need to accelerate the construction of an emergency language service talent system to cultivate composite language talents. In this context, the vocational colleges and universities should pay attention to develop emergency language majors, establish emergency linguistics and add emergency language courses as soon as possible (Li et al., 2020). From the above, it can be seen that both in China and internationally, there is a focus on emergency language education and the cultivation of emergency language talents, but at present, the development of emergency linguistics majors in Chinese universities is still in its infancy, the emergency language courses have not yet been launched (Sun & Zheng, 2022). On this basis, this study implemented an innovative design of an emerging course on emergency language in the post-epidemic context, which not only helps to expand the teaching content in different contexts, but also has great significance for the improving national language service capabilities.

The theoretical framework of scaffolding, as proposed by Wood et al. (1976), has been uncritically extrapolated from its original socio-constructivist roots into educational pedagogy, often without rigorous empirical validation. While Van de Pol et al. (2010) and Puntambekar (2022) assert its efficacy in metacognition and classroom engagement, these claims gloss over two critical gaps: (1) the assumption that teacher-led scaffolding universally benefits all learners, neglecting cultural, cognitive, and socio-economic disparities that may mediate its effectiveness; (2) the conflating of correlation with causation—active participation does not necessarily equate to deeper learning outcomes. Zhu’s (2025) observation further exposes a methodological flaw in hybrid education research: the proliferation of studies has not translated into scaffold designs that address the asymmetry of digital and in-person learning modalities. The field remains mired in anecdotal success stories, with scant attention to longitudinal data or control-group comparisons. A critical re-examination of scaffolding’s theoretical boundaries—and its empirical limitations—is overdue, therefore, based on constructivist learning theory, this study set up an experimental group and a control group in an emergency language course, implemented the scaffolding in the experimental group and the traditional teaching methods in the control group. At the same time, comparing the perceived teacher support behavior in both groups' of classrooms is used to detect students' psychological processes and learning outcomes.

Self-regulated learning (SRL) is a positive, goal-oriented process in which students set their own learning goals and dynamically monitor, regulate and control their cognitive, motivational, and behavioral based on environmental needs (Dent & Koenka, 2016). In recent years, researchers have begun to focus on the association between SRL and learners' learning outcomes based on achievement motivation theory (Urdan & Kaplan, 2020). Scholars have proposed different models of SRL and believed that SRL is a key competency for learners, and the self-regulation process is also seen as a key factor that can lead to better self-directed processes for learners (Adam et al., 2017; Li et al., 2020; Wong et al., 2019a, 2019b). However, it is a challenge for learners to acquire sufficient self-regulation skills, which requires teachers to monitor each student' s self-regulation process and provide appropriate scaffolding in a timely manner (Song & Kim, 2021). Therefore, the role of SRL in learning outcomes is related to the effective support provided by teachers via scaffolding (Granberg et al., 2021). Synthesizing previous results, it was found that periodicity is a key component of SRL (Qi et al., 2025) and that the periodical SRL model consists of different types and subprocesses, but few studies have focused on each type (Zeidner & Stoeger, 2019). Thus, based on expected value theory, this study used the definition of the three types of SRL proposed by Hong et al. (2021)—preparation, performance, and self-evaluation—to observe the effects of teacher scaffolding support on emergency language course learning outcomes under the three types of students' SRL, ultimately to propose specific instructional strategies in response to the findings.

## Research Model and Hypotheses

### Research Model

**Fig. 1:**
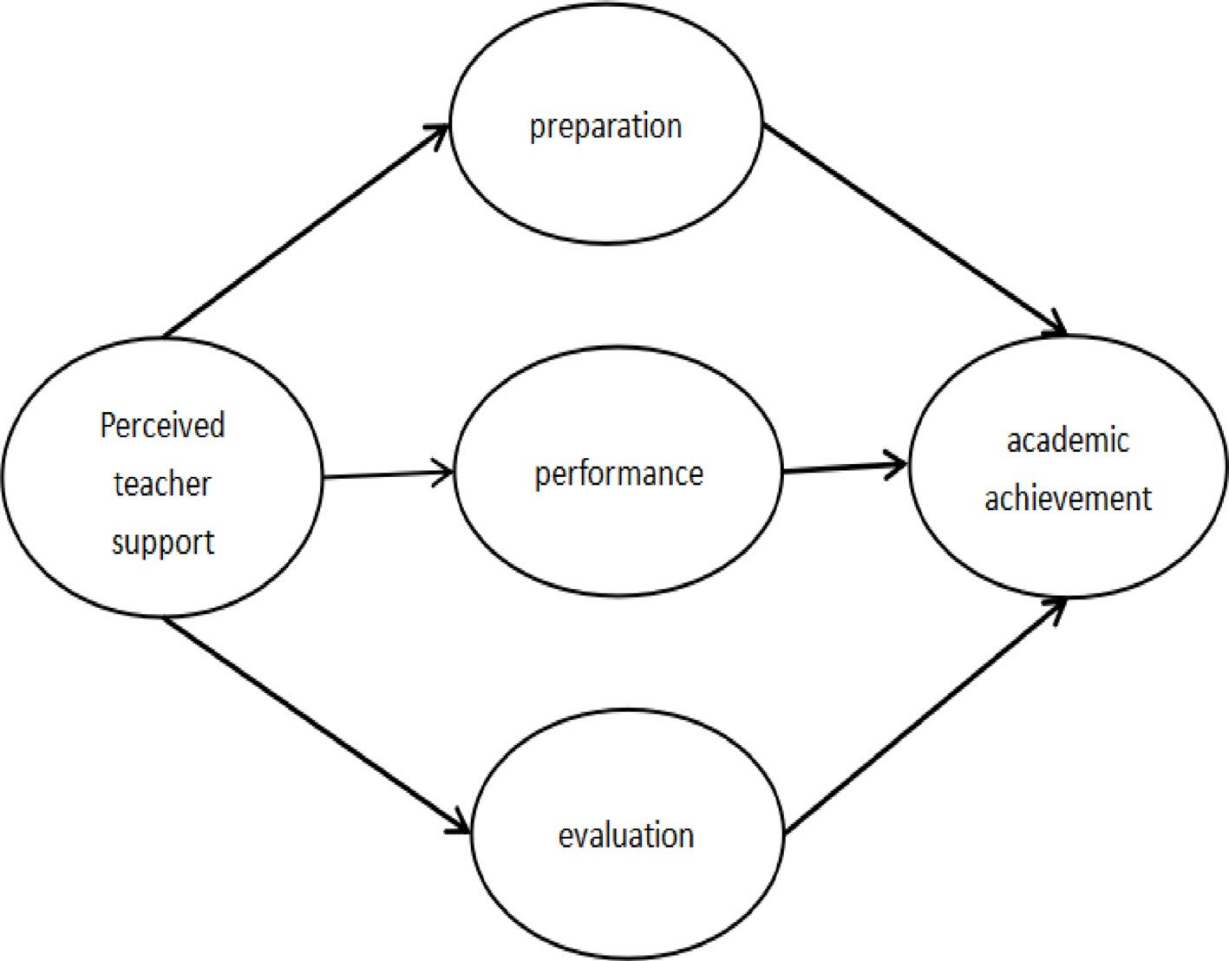
The Theoretical Model

Based on constructivist learning theory and expected value theory, when students lack strong foundational skills, giving them little support can cause cognitive overload and confusion, so that scaffolding provides strong support for students and has an impact on their self-regulation (Howe, 2013; Kirschner et al., 2006). Based on the viewpoint of achievement motivation and SRL model, the stronger the motivation to learn, the more positive the achievement behavior will be, and the better the perceived value will be (Hong et al., 2021). SRL further promotes students' academic achievement (Zimmerman, 2020). Therefore, this study proposed eight research hypotheses and constructed a theoretical model based on the three types of SRL, as shown in Fig. 1.

### Research Hypotheses

#### The Relationship Between Perceived Teacher Support and Self-Regulation

Based on the self-determination theory of motivation continuum model (Deci & Ryan, 2000), this study reconstructs teacher support behavior into three types of scaffolds: autonomy support, ability support and relationship support. The data indicates that teacher support can enhance intrinsic motivation by meeting students' basic psychological needs (Adams et al., 2017), and Brenner (2022) also obtained the same cross-culturally validation in a meta-analysis. Furthermore, Wang et al. (2024) revealed in path analysis that teacher support influences SRL through the mediating chain of motivation. That is, teacher ability support can enhance students' academic self-efficacy, thereby enhancing cognitive strategy use. This finding is consistent with the social motivation model, which suggests that teacher behavioral cues (such as timely feedback) indirectly regulate SRL behavior through students' perceived supportive environment (Wentzel et al., 2016). Reeve et al. (2002) further confirmed that when teacher support matches students' positive emotions, a positive cycle of “support—positive emotions—academic performance” can be formed. In summary, this study proposes a dual pathway mechanism: teacher support can both strengthen students' intrinsic motivation and indirectly shape SRL behavior through environmental perception, both of which jointly promote academic performance.

Based on the above theories and studies, this study concluded that teacher support can facilitate SRL behavior. Because some scholars have suggested that attention should be paid to the complex internal structure of SRL (Zeidner & Stoeger, 2019), this study used the definition of three types of SRL proposed by Hong et al. (2021)—preparation, performance, and self-evaluation—to propose the following hypotheses:

##### H1:

Perceived teacher support is positively related to preparation.

##### H2:

Perceived teacher support is positively related to performance.

##### H3:

Perceived teacher support is positively related to self-evaluation.

#### The Relationship Between the Three Types of SRL and Learning Achievement

This study used the three types of SRL—preparation, performance, and self-evaluation—as a cyclical process to explore college students' SRL in the emergency language classroom. Preparation refers to the emotional regulation and environmental construction of the learner's participation in the task, which have beneficial effects on the learner's emotional adjustment and activation of pre-reflection during the course (Hong et al., 2021). This is followed by performance, whereby learners will complete the program by exercising self-control and self-observation (Wong et al., 2019a, 2019b), including task strategies and time management (Hong et al., 2021), which can help them control their emotions, attention, and environment and adapt to environmental changes (Ridgley et al., 2020). In self-evaluation, learners reflect on their performance stages and form new goals and plans (Wong et al., 2019a, 2019b), which is very effective for forming learning strategies. In college, students' SRL is a predictor of their academic performance and an important condition for good academic performance (Schunk & DiBenedetto, 2020), SRL processes have been shown to be effective in terms of promoting the academic achievement (Michalsky, 2020). Zimmerman et al. (2001) suggested that process goals significantly enhance learners' self-regulatory beliefs, which are more effective than outcome goals. Therefore, this study infers that under adaptive scaffolding conditions, students exhibit self-regulated behaviors and their knowledge systems will significantly improve. Based on the above theories and studies, this study proposed the following hypotheses:

##### H4:

Preparation is positively related to academic achievement.

##### H5:

Performance is positively related to academic achievement.

##### H6:

Self-evaluation is positively related to academic achievement.

#### The Relationship Between Perceived Teacher Support, SRL and Learning Achievement

The ability development of SRL is essentially a social cognitive process (Zimmerman, 2000), and its effectiveness is highly dependent on the structural support provided by teachers (Wong et al., 2019a, 2019b). According to social cognitive theory (Bandura, 1986), teacher feedback and prompts are not only external interventions, but also key mediators for learners to internalize metacognitive strategies. When students lack accurate self-monitoring abilities, their learning performance is significantly lower than theoretical predictions (Dunlosky & Rawson, 2012). The dynamic scaffolding theory proposed by Van de Pol et al. (2010) further elucidates that the effectiveness of teacher support needs to be achieved through goal-directed adaptive intervention, which is in line with feedback loop model (Wilson & Devereux, 2014). Similarly, Azevedo and Hadwin (2005) found in SRL meta-analysis that when teacher scaffolding directly targets metacognitive deficits, its promoting effect on learning outcomes conforms to the positive cycle hypothesis of “self-efficacy performance” in social cognitive theory. Therefore, this study concluded that not only does perceived teacher support have a positive direct effect on learning achievement, but it also has an indirect effect on learning achievement through the mediating effect of SRL; the following hypotheses were therefore proposed:

##### H7:

SRL mediates the effect of perceived teacher support on learning achievement.

##### H8:

In the emergency language course, students learning with scaffolding would show significantly higher learning achievement than those learning with traditional instruction.

## Research Design

### Course Design

The emergency language course was designed based to the Content and Language Integrated Learning (CLIL) method, focusing on cultivating students' emergency language proficiency, the awareness of emergency language services and the professionalism as emergency personnel in the context of both language and culture (Xi & Liu, 2020). This content of this course refers to the idea of “Concise Chinese for epidemic prevention and control,” issued by the Ministry of Education (2016) to use plain English as the teaching content, aiming to enable students to use concise, clear and practical English to communicate linguistically when major public emergencies occur.

This study used a quasi-experimental design with scaffolding instruction and traditional instruction for the experimental and control groups respectively for eight weeks, with three hours of instruction per week for each group. The course content was based on the classification of emergency events, which divided emergency events into four teaching units: natural disasters, accidents and disasters, public health events and social security events. In the first week, the content was the first unit on emergency language in the context of public health emergencies, assuming that the language target was foreigners during the COVID-19 epidemic, and the task was how to communicate with different groups of people, such as ordinary people, people who go out, people who are isolated at home, and people with special occupations. The goal was to help students master the English language for emergency situations and become proficient in translating and verbalizing the methods and procedures used to prevent and control the COVID-19 epidemic. In the second and third weeks, the learning content was the emergency language for natural disasters. This unit used floods, earthquakes, or other severe weather as a context for learning vocabulary and sentences, and focused on how to communicate with foreigners in the face of disasters. In the fourth and fifth weeks, the learning content was the emergency language for social security emergencies. This unit used situations such as terrorist attacks and refugee arrivals to learn relevant vocabulary and to envision how to handle emergencies and communicate appropriately in the aftermath when facing foreigners. The sixth and seventh weeks focused on sudden accidents and disasters. This unit used social emergencies such as plane crashes and train derailments as contexts to help students learn how to communicate with foreigners about the handling of such events and emergency interventions. The 8-week was devoted to student presentations, teacher assessment and feedback, and students voluntarily completed a post-test and a questionnaire after class. The content of this study was confirmed by two foreign language teaching experts and four English teachers as being reasonable and scientific.

### Scaffolding Instruction in Emergency Language Courses

**Fig. 2:**
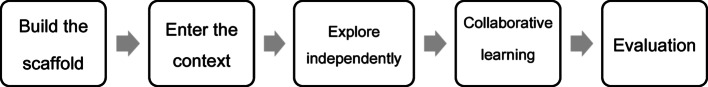
Scaffolding process

According to He (1997), scaffolding consists of five components: build the scaffold, enter the situation, independent exploration, collaborative learning, and evaluation. It has been established that teacher support in scaffolding enables students to perceive support for self-regulation, and metacognitive support in turn affects academic performance. Therefore, this study applied to the scaffolding model to the emergency language course to design the curriculum in the experimental group, which was designed as follows: (1) Build the scaffolding: in this stage teachers design different learning units based on the content that students have already learned. The teacher determines the learning situation through pre-testing. (2) Enter the context: the teacher uses movies or pictures to create a public emergency situation to stimulate learners' interest in learning, while guiding them to find problems, analyze them and explore the solution paths, and further participate in the classroom. (3) Explore independently: the teacher observes the learners' reactions to the problems as they complete the tasks, and provides the principles and methods of problem solving at the right time. The teacher's guidance gradually decreases as the students' problem-solving ability increases until the students are able to complete the tasks independently. (4) Collaborative learning: the learners unify their opinions through group work and discussion, and gradually clarify the concepts, forming a correct and comprehensive understanding under the teacher's tips and guidance, and finally completing the construction of knowledge. (5) Evaluation: lastly, students present their learning results in various forms. Teachers should provide timely feedback and evaluation at the same time. The specific process is shown in Fig. 2.

The control group used traditional language teaching methods, namely lecture based teaching, to teach emergency language courses. In traditional classrooms, teachers first need to present the knowledge points of emergency language in the pre-class stage, especially focusing on teaching emergency terminology, phrase combinations and grammar in both Chinese and English; Then take students to practice in class, repeatedly practicing and strengthening the content taught by the teacher in the pre-class stage; In the post class stage, the teacher organizes students into groups and assigns different assignments to each group, allowing them to take turns presenting their homework results on stage, which is the output of traditional teaching methods.

## Research Process and Data Collection

**Fig. 3:**
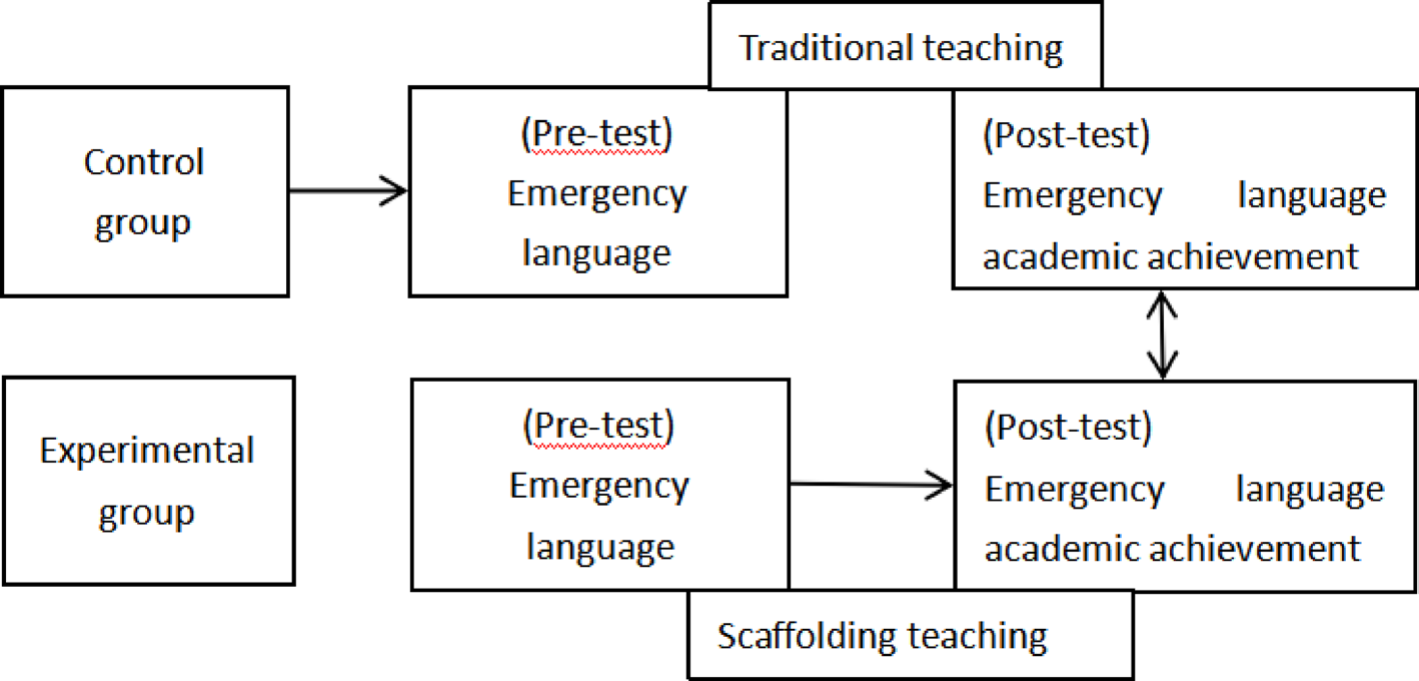
Experimental research process

In this study, a quasi-experimental design was used to evaluate the effectiveness of teaching (Williams et al., 2022). This study used convenience sampling to invite 225 s-year students of a Chinese general university majoring in teaching Chinese to speakers of other languages were invited to participate in the experiment in an 8-week emergency language course. The researcher divided the students into an experimental group of 115 students and a control group of 110 students. The experimental group was taught using scaffolding while the control group was taught in a traditional manner. At the beginning of the experiment, the teacher explained the content and objectives of the course to the students, and administered a closed-book pre-test on the students' emergency language skills of two groups of students. After the experiment, the researcher administered a post-test and used a questionnaire survey for students' self-evaluation. The process of the experimental study is shown in Fig. 3.

### Participants

The 225 participants were all second-year students of teaching Chinese to speakers of other languages, aged 18–20, with similar learning backgrounds. This major was chosen to participate in the experiment because many studies have focused on its close relationship with public emergencies (Cui, 2020). 8 invalid data were removed, leaving a total of 217 valid participants, including 112 in the experimental group and 105 in the control group. Using stratified random grouping, the experimental group of 112 people was divided into two classes of 56 people each, while the control group of 105 people was divided into two classes of 55 and 50 people. Each class teaches separately on the same day, with the same teaching staff, teaching content and teaching arrangements.

### Research tools

Self-evaluation is widely used in the field of foreign language teaching, referring to learners' evaluation of their own knowledge and skills (Little, 2005). Numerous studies on self-assessment of language proficiency by foreign language learners have shown that self-assessment is an effective and reliable way to measure one's own language ability (Ünaldı, 2016), and learners participate in self-assessment which can produce precise results (Little, 2005).

#### Perceived Teacher Support

This study used the learning climate questionnaire (LCQ) to examine students' perceptions of teacher support (Williams & Deci, 1996), which containing 15 items.

#### Self-Regulated Learning

The SRL scale was adapted from the self-regulated learning scale by Hong et al. (2021) to assess participants' SRL in the experimental classroom. The SRL scale includes 24 items and three dimensions: the preparation dimension, the performance dimension and the assessment dimension. Because the original scale measured the online learning process during the COVID-19 epidemic, the scale items were reduced.

#### The Pre-test and Post-Test Questions

The pre-test and post-test questions of this study are from internationally recognized test questions, which are the "Emergency Disaster Preparedness Exam Questions" developed by the "Language Concept of English and Spanish" organization. There are a total of 30 question types, and the language of the questions is English.

## Results and Discussion

### Item Analysis

SPSS 22.0 was used for item analysis. After an independent sample *t* test was performed for each item, the results showed that the composite reliability (CR) of each item was between 4.35 and 9.33. The CR greater than 3 indicates that the questionnaire has good discrimination (Carmines & Mclve, 1981). The factor loading (FL) of more than 0.5 should be used as an indicator of internal validity (Hair et al., 2013). According to this criterion, the perceived teacher support dimension was reduced from 13 to 12 items, the preparation of SRL dimension was reduced from five to four items, and the performance dimension was reduced from seven to six items. The items were further analyzed using the maximum variance rotation method; the rotated component matrix showed good factor loadings for the four dimensions, as shown in Table 1.

**Table 1:** Item Analysis Table

Dimension	FL	Maximum variance rotation	
		CR	Explanation rate
Perceived teacher support	0.489–0.866	6.307	23.30%
Preparation	0.485–0.547	7.518	12.34%
Performance	0.484–0.801	7.262	20.85%
Self-evaluation	0.589–0.882	6.377	10.11%

### Reliability and Validity Analysis

Hair et al. (2013) suggested that Cronbach's alpha should be greater than .70 to be considered as passing the reliability test. The questionnaire comprised four dimensions: perceived teacher support with 12 questions, α = .96, and self-regulated learning including three dimensions: the preparation dimension, α = .87, the performance dimension, α = .91, and the assessment dimension, α = .92. The CR of all the dimensions met the recommended criteria, indicating that the questionnaire had good reliability.

The FL greater than 0.500 is required for convergent validity (Hair et al., 2013). The results of the study showed that FL ranged from 0.652 to 0.866 for the perceived teacher support dimension, 0.508 to 0.655 for the preparation dimension, 0.503 to 0.801 for the performance dimension, and 0.589 to 0.882 for the self-evaluation dimension of SRL. In addition, Fornell and Larcker (1981) suggested that when the average variance extracted (AVE) of the dimension is less than 0.500 but the CR is higher than 0.600, the dimension validity is still sufficient, so the results of this study also met the suggested criteria. The results are shown in Table 2.

**Table 2:** Dimension reliability and validity analysis

Dimension	*M*	*SD*	α	FL	CR	AVE
Perceived teacher support	4.145	0.631	0.96	0.652–0.866	0.917	0.561
Preparation	4.200	0.549	0.87	0.508–0.655	0.739	0.417
Performance	4.168	0.511	0.91	0.503–0.801	0.885	0.528
Self-evaluation	4.243	0.532	0.92	0.589–0.882	0.906	0.580

The pre-test and post test questions are the "Emergency Disaster Preparedness Exam Questions". There are a total of 30 question types, and the language of the questions is English. According to the calculation method of the overall CVI index of expert validity, the review results of 4 experts were summarized, and the CVI index of 30 items reached .833, indicating good expert validity.

### Research Model Validation Analysis

**Fig. 4:**
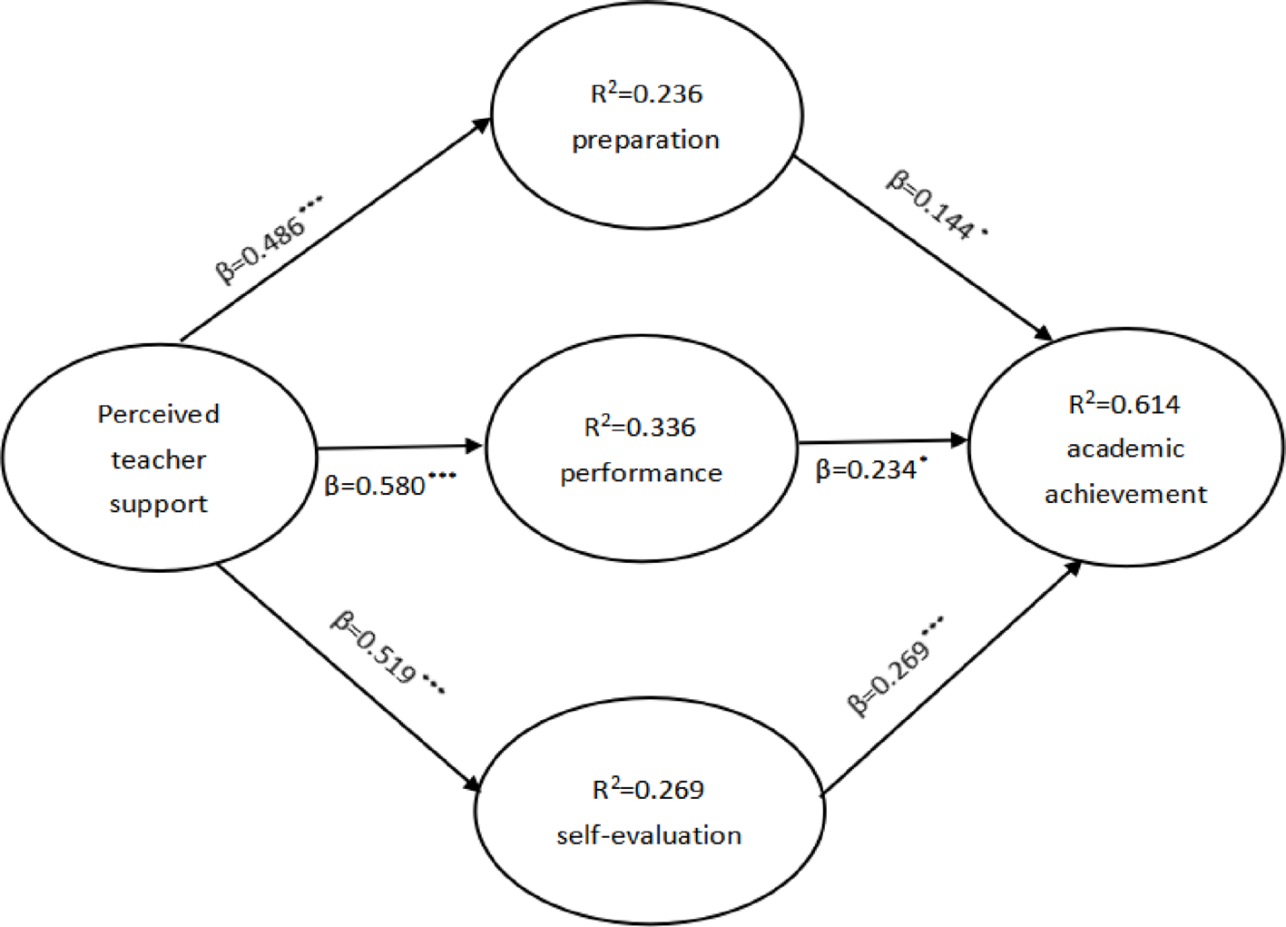
Research Model Validation. ***p < 0.001, **p < 0.01, *p < 0.05

The model validation results showed that perceived teacher support showed a positive correlation with preparation (*β* = 0.486,* p* < 0.001), with an explanatory power of 23.6%; perceived teacher support showed a positive correlation with performance (*β* = 0.580, p < 0.001), with an explanatory power of 33.6%; perceived teacher support showed a positive correlation with self-evaluation (*β* = 0.519, *p* < 0.001), with an explanatory power of 26.9%. Self-regulated learning preparation type showed a positive correlation with learning achievement (*β* = 0.144, *p* < 0.05), performance showed a positive correlation with learning achievement (*β* = 0.234, *p* < 0.05), and self-evaluation showed a positive correlation with learning achievement (*β* = 0.466, *p* < 0.001); the explanatory power of the three types of self-regulated learning for learning achievement was 61.4%. Thus, this study H1, H2, H3, H4, H5 and H6 were all validated, as shown in Fig. 4.

### Homogeneity Testing

Before conducting the teaching experiment, it is necessary to verify the experimental group and control group of the emergency language course, and test whether the experimental group and control group of the research sample have homogeneity (Creswell, 2012). In this study, an independent sample *t*-test was used, and if the *p*-value is greater than .050, it indicates homogeneity between the two groups (Glass & Hopkins, 1996), and the teaching experiment can be implemented. The results of the independent sample* t*-test analysis showed that the average values of the questionnaire survey and test results were relatively close between the control group and the experimental group, and the *p*-value was not significant. This indicates that there is no statistically significant difference in emergency language proficiency between the two groups during the pre-test, and they have homogeneity which is a prerequisite for teaching experiments. Please refer to Table 3 for details.

**Table 3:** Homogeneity testing

Project	Group	*n*	*M*	*SD*	***t***	***p***
Pre-test	EG	112	36.749	6.134	.675	.937
	CG	105	35.842	6.387		
Questionnaire	EG	112	21.449	3.208	1.256	.217
	CG	105	22.309	3.716		

***p < 0.001, **p < 0.01, *p < 0.05

### Mediator Effect

The results of the mediation model validation are shown in Table 4: perceived teacher support had a significant effect on learning achievement (*β* = 0.557, *p* < 0.001); self-regulated learning had a significant effect on learning achievement (β = 0.577, *p* < 0.001); perceived teacher support (*β* = 0.159, *p* < 0.05) and self-regulated learning (*β* = 0.690, *p* < 0.001) had a significant effect on learning achievement. Among them, the predictive effect of perceived teacher support on learning achievement decreased from 0.557 to 0.159, so SRL had a partial mediation effect between perceived teacher support and learning achievement; hypothesis of H7 was therefore valid.

**Table 4:** The mediation effect

Variables	Learning achievement (*β*-value)		SRL (*β*-value)	Learning achievement (*β*-value)
Perceived Teacher support	0.557***		0.577***	0.159*
SRL				0.690***
△*R* ^2^	0.303		0.326	0.627
F-value	46.667 (0.000)		51.805 (0.000)	86.691 (0.000)

*p < 0.05 **p < 0.01 ***p < 0.001.

### Variance Analysis

In order to demonstrate whether there was a difference in language proficiency between the two groups before the experiment, a closed-book pre-test was administered to the students of both groups on their emergency language proficiency before the experiment. The content of the pre-test paper was the same for both groups, and the test lasted 40 min. The average scores of the pre-test were 44.97 and 45.08 for the control group and the experimental group, which were close to each other, indicating that there was no significant difference between the two groups in terms of emergency language proficiency. The results illustrate that the objective conditions and requirements of the quasi-experimental design were met. After the 8-week experiment, a post-test was administered to both groups to determine the effectiveness of the scaffolding and traditional instruction in the emergency language course. The post-test questions were the same as the pre-test questions, but the order of the questions was switched. The post-test scores of the control and experimental groups were subjected to independent samples *t* test and were statistically analyzed using the SPSS software. The *p*-value was 0.000 (*p* < 0.001) and the *t*-value was -4.942, which proved that the experimental group had a more significant increase in the post-test scores compared to the control group. Due to the short duration of the experiment, Cohen's *d*-value was 0.680, which was a small effect, but it confirmed H8; that is, the scaffolding in emergency language courses is more effective than traditional instruction in terms of promoting students' learning achievement. The statistical results are shown in Table 5.

**Table 5:** Comparison of the post-test scores of the control and experimental groups

Test	Group	*M*	*SD*	*t*	*p*	*d*
Post-test	Control group	55.59	7.762	-4.942	0.000	0.680
	Experimental group	60.87	7.764			

## Research Discussion

### Perceived Teacher Support Showed a Positive Correlation with the Three Types of SRL; Three Types of Students' SRL Showed a Positive Correlation with Learning Achievement

SRL plays a critical role in assessing student learning achievement, which is related to the effective support provided by teachers. Scaffolding implies that teachers can provide support to learners to address their metacognitive deficits and thereby improve their knowledge acquisition and other skills (Huang et al., 2021). In the current study, students' perceived teacher support in the emergency language classroom was positively correlated with their preparation, performance, and self-evaluation; in other words, the greater the students' perceived teacher support, the more pronounced their SRL behaviors and characteristics. Many studies have shown that there is a significant correlation between SRL and academic achievement (Cicchinelli et al., 2018). In this study, we have validated the above viewpoint and conducted a process study on SRL, concluding that all three types of students' SRL were positively associated with learning achievement; that is, the preparation, performance, and self-evaluation of students were positively associated with their learning achievement. It can be seen that in the emergency language classroom, scaffolding implies high teacher support, and students' perceived teacher support influences the three types of SRL, which ultimately has a positive effect on learners' achievement. Given the significant impact of SRL on academic performance (Zimmerman, 2020), the education department should prioritize incorporating SRL skills into curriculum standards and strengthen training on specific strategies for teacher support, such as promoting self-regulation cycle model through teacher workshops. The predictive effect of SRL on academic performance is valid in different cultural backgrounds (Wang et al., 2013). Although the sample comes from China, the research results are consistent with the findings of European and American scholars in cross-cultural research on SRL (OECD, 2022), indicating that there is a global need to pay attention to metacognitive cultivation.

### SRL Partially Mediates the Effect of Perceived Teacher Support on Learning Achievement

The results of this study suggest that academic achievement can influence perceived teacher support by enhancing students' SRL abilities. The SRL strategies are part of the SRL process, and the application of SRL strategies often predicts academic achievement (Wang et al., 2013). Therefore, in this experimental emergency language course, the teachers implemented SRL strategies through a scaffolding approach to achieve the goal of influencing academic achievement. This discovery provides key insights for education policies, namely that SRL strategies not only rely on individual student abilities, but also require teachers to form a virtuous cycle through dynamic support such as goal setting and feedback adjustment.

### Scaffolding in the Emergency Language Classroom is Significantly more Effective Than Traditional Teaching

For language learners, scaffolding allows activities to be designed for language practice. Compared to traditional teaching methods, scaffolding can help students to fully participate in the classroom and improve their foreign language skills (de Oliveira, 2016). The results of this study also showed that students in the experimental group with the same teacher, the same number of hours and the same content had significantly higher learning achievement compared to those learning via traditional teaching, suggesting that scaffolding is more effective than traditional teaching for foreign language instruction. This study of an instructional design for an emerging emergency language course validates the high support and challenge of the scaffolding interventions in empirical study, and enriches the possibilities for language instructional course design. Thus, it is recommended that other foreign language teachers try to change their consistent approach of comprehension, interpretation, and meaning formation by designing foreign language teaching courses, focusing on learner participation to enhance learning achievement.

## Research Limitations and Future Recommendations

First, emergency languages include standard Mandarin, foreign languages, dialects, ethnic languages, sign languages, and so on, but only a foreign language was studied in this research; therefore, subsequent studies can expand the types of languages and explore the effectiveness of other languages under the scaffolding design. Second, the participants in this study were college students with the same knowledge background. Attention should be paid to expanding the subject areas of the participating samples in the follow-up studies in order to achieve the goal of using various majors to conduct emergency language education in colleges and universities. Finally, the integration of online foreign language courses with scaffolding in the post-epidemic era could be the next research goal.

## Declarations

### Additional Declaration

The authors inadvertently made a duplicate submission in the past and posted a preprint. This matter has been explained to the editorial office.
